# Multi-attention cross-scanning VM-UNet for X-ray welding defect detection of steel pipeline

**DOI:** 10.1371/journal.pone.0341805

**Published:** 2026-02-06

**Authors:** Ting Zhang, Dengwu Wang, Shanwen Zhang

**Affiliations:** School of Computer Science, Xijing University, Xi’an, China; Zhengzhou University, CHINA

## Abstract

Welding defect detection of steel pipelines (SPWDD) is critical for ensuring the safe use of oil/gas pipelines. Due to the complex morphology of welding defect X-ray images (WDXI) in steel pipes and the/low contrast between the defects and the background, SPWDD is an important and challenging topic. To address these challenges, a Multi-Attention Cross-scanning VM-UNet (MACVM-UNet) for SPWDD is constructed. This model adopts the cross-Scanning Visual State Space Model (CVSS) to capture the local features and long-range dependencies, introduces channel attention skip connections (CASC) instead of the conventional skip connections to enhance the performance of globally and locally feature fusion, and employes the multi-scale Attention Feature Aggregation (MSAFA) module to fuse the multi-scale features. The combination of CVSS, CASC and MSAFA can effectively enhance the performance to extract the global-local features of small-sized and large-sized WDXIs. The experimental results on the WDXI dataset validate that the proposed MACVM-UNet outperforms the state-of-the-art models with superior SPWDD performance while maintaining low computational complexity. It achieves the *mAcc* 86.03%, *mPre* of 86.14% and mF1 of 84.6% with lower training time of 1.61 h. The proposed method provides an efficient and feasible solution for non-destructive SPWDD of oil/gas pipelines.

## 1. Introduction

The oil/gas pipeline network plays a vital role in the modern economy, yet its operation under high-pressure conditions carries significant risks, with welding defects being a leading cause of pipeline failure. Welding defect detection of steel pipelines (SPWDD) is critical for ensuring structural integrity and safe operation [1]. This task is particularly challenging due to the complex, diverse, and often low-contrast appearance of defects in X-ray images (WDXIs) [2].

Traditional machine learning methods for SPWDD rely on handcrafted feature extraction, which is inadequate for the complexity of welding images, limiting their suitability for automated detection [3,4]. Consequently, deep learning, especially Convolutional Neural Networks (CNNs) like U-Net and YOLO variants, has become predominant, achieving notable success [5]. However, CNNs are inherently limited by their local receptive fields, which hinder their ability to model global context and long-range dependencies within an image, often leading to suboptimal segmentation or detection of defects with intricate or extended morphologies.

Transformers and Vision Transformers (ViTs) have emerged as powerful alternatives, excelling at capturing global information and long-range dependencies through self-attention mechanisms, and have shown promise in SPWDD tasks [6]. Wang et al. [7] presented an innovative approach for detecting defects in insulators used in high-voltage power transmission lines, employing an enhanced Detection Transformer model (IF-DETR), and then developed a new hybrid architecture model for IC surface defect detection based on ResNet and ViT(ResNetViT) [8]. Despite their effectiveness, the quadratic computational complexity of self-attention with respect to image resolution (O(n²)) severely limits their scalability and practical application for processing high-resolution WDXIs.

To address the shortcomings of CNN and Transformer, the visual state space model (VSS) has been presented. VSS inherits the core strength of Mamba in sequence modeling and extends it to two-dimensional image data [9,10]. The global context of the entire image with linear complexity is modeled through its state space formula and selection mechanism, while dynamically adjusting the weight distribution of important features. This feature makes it theoretically highly suitable for intensive prediction tasks such as image segmentation and defect detection, as it avoids the limitations of CNNs in modeling remote dependencies while overcoming the low computational efficiency and local detail capture bottleneck of ViTs [11,12]. However, how to effectively combine the efficient global modeling ability of VSS with the precise perception of local image structures (such as edges and textures) to further improve the segmentation accuracy remains an important research gap. To bridge this gap, a Multi-Attention Cross-scanning VM-UNet (MACVM-UNet) for SPWDD is proposed. The model is designed to synergistically capture comprehensive global dependencies and detailed local features with efficient computation. The key innovations that address the identified limitations are:

(1) Cross-scanning VSS(CVSS) Module. The standard VSS block is replaced with a CVSS module that employs scanning along four distinct paths (horizontal, vertical, diagonal, and anti-diagonal) to ensure more comprehensive global context aggregation, mitigating the directional bias of single-path scans.
(2) Channel Attention Skip Connection (CASC). The standard skip connections in U-Net are substituted with CASC modules to adaptively recalibrate and fuse features from the encoder and decoder, enhancing feature complementarity and reducing semantic gaps, thereby improving the reconstruction of defect details.
(3) Multi-scale Attention Feature Aggregation (MSAFA) Module. MSAFA is integrated into the bottleneck, employing dilated convolutions and dual-attention (spatial and channel) to effectively aggregate and refine multi-scale features from the encoder, strengthening the model’s ability to represent defects of varying sizes and morphologies.

The remainder of this article is outlined as follows. Section 2 briefly introduces the relevant work. Section 3 details the architecture of MACVM-UNet and its main components. Section 4 presents the experiments and results of SPWDD on the WDXI dataset. Finally, Section 5 simply summarizes the research results of this study and the future work.

## 2. Related work

Recent advancements in SPWDD are primarily driven by deep learning, which can be categorized into DL-based methods and the emerging Mamba-based methods [1,9,13].

### 2.1 DL-based methods

Hou et al. [14] comprehensively summarized the research on automatic SPWDD from defect preprocessing, defect segmentation, defect classification, application, limitations and challenges of DL-based methods. Wang et al. [15] reviewed the automatic SPWDD methods from X-ray images, including the traditional ML-based and DL-based methods from datasets and network structures, welding defect segmentation and classification, and the techniques used to solve the problems of small datasets. DL-based SPWDD approaches have evolved from CNNs to Transformers. Table 1 outlines their main characteristics and limitations.

**Table 1:** Summary of DL-based Methods for SPWDD.

Category	Representative models/Techniques	Key advantages for SPWDD	Main limitations
CNN-based	U-Net, YOLO series [16], Attention U-Net (AU-Net) [17]	Strong local feature extraction; Good for texture and shape.	Limited receptive field; Weak at modeling long-range dependencies.
Transformer and ViT-based	CGTD-Net [18], ViT, TransUNet [19], RT-DETR [20], ResNetViT[7], IF-DETR [8]	Excellent global dependency modeling; Powerful for complex scenes.	Quadratic computational complexity (O(n²)); High resource demands for high-res images.
Others	Various customized CNN-Transformer hybrids	Attempt to balance local and global feature capture.	Often inherit complexity or design trade-offs from base architectures.

From the above analysis, it is known that CNNs and ViTs can improve the performance of SPWDD, but they still have some limitations, such as CNNs are limited to model the global and geometric features, while the quadratic computational complexity of ViTs (*O*(*n*²)) severely limits their scalability for high-resolution SPWDD tasks.

### 2.2 Mamba-based methods

Recently, Mamba has become a breakthrough in the realm of DL, and has immense potential in modeling global context with linear computational complexity, addressing the main limitation of Transformer and ViTs while maintaining superior long-range modeling capabilities [9,10]. Bo et al. [21] provided the first systematic review of Mamba-based methods, analyzed approximately 120 studies, and constructed a comprehensive classification. The introduction of VSS with linear complexity has led to a new paradigm. Table 2 outlines the evolution of visual Mamba models.

**Table 2:** Evolution of VMamba models.

Category	Core mechanism	Representative models/Techniques	Key advantages for SPWDD
CNN-based	Local feature extraction via convolutional kernels.	U-Net, YOLO series, Attention U-Net (AU-Net) [17]	Strong local feature extraction; Good for texture and shape.
Transformer-based	Global context modeling via self-attention.	Vision Transformer (ViT), TransUNet [19], CGTD-Net [18], RT-DETR [20]	Excellent global dependency modeling; Powerful for complex scenes.
Others	Hybrid or specialized architectures.	Various customized CNN-Transformer hybrids	Attempt to balance local and global feature capture.

VMamba extends Mamba to 2D vision tasks by scanning 2D images in both horizontal and vertical directions, while it cannot comprehensively handle spatial regions in a single scan [22]. VM-UNet is a visual Mamba U-Net. It introduces VSS as the basic block for capturing context information and constructs an asymmetric encoder-decoder structure with fewer convolutional layers to save computational costs [10]. MSVM-UNet is a multi-scale VM-UNet model [23]. It can effectively capture and aggregate multi-scale features from the hierarchical features of the VMamba encoder and better handle two-dimensional visual image data. LightM-UNet is a lightweight Mamba-UNet model [24]. It integrates Mamba and U-Net in a lightweight framework, extracts deep semantic features using the residual VMamba layer, and models long-range spatial dependencies with linear computational complexity. Local-mamba adopts the local scanning strategy to effectively capture the local dependencies while maintaining a global perspective [25].

### 2.3 Summary of MACVM-UNet

Inspired by the above modified Mamba models, a Multi-Attention Cross-scanning VM-UNet (MACVM-UNet) for SPWDD is constructed, and is demonstrated by extensive experiments on the WDXI dataset. As summarized in Table 3, existing Mamba-based models have made significant strides but leave room for improvement in comprehensive feature capture and fusion. MACVM-UNet is designed to address these aspects synergistically. Table 3 shows the comparison table to synthesize information and visually underscore the distinctive features of MACVM-UNet.

**Table 3:** Comparison of the existing models and MACVM-UNet.

Method category	Core mechanism	Main advantages	Main limitations	Relation to MACVM-UNet
Traditional & ML	Handcrafted features + classifiers.	Interpretable; low data requirement.	Poor generalization; inadequate for complex defects.	Baseline, highlighting the need for deep feature learning.
CNN	Convolutional Neural Networks, encoder-decoder.	Strong at capturing local textures/shapes.	Limited receptive field; weak long-range dependency modeling.	Provides the foundational U-Net architecture that MACVM-UNet enhances.
Transformer	Self-attention for global context.	Excellent global dependency modeling.	Quadratic computational complexity O(n²); high memory cost.	Motivates the search for efficient global modeling (e.g., Mamba).
For Class Imbalance [7]	DL strategies for imbalance (re-weighting, etc.).	Directly tackles data imbalance.	May be highly tailored to a specific domain.	Informs the design for robustness under imbalanced distributions.
For Multi-Scale [8]	Multi-scale feature fusion, attention.	Explicitly handles large size variance.	May increase model complexity.	Validates the necessity of the MSAFA module.
VM-UNet Family [22]	SSMs, linear-complexity modeling.	Linear complexity; global modeling capability.	Early scans may have directional bias; suboptimal multi-scale fusion.	Direct precursors & targets for improvement.
MSVM-UNet [23]	Multi-scale convolution inside VSS block.	Introduces multi-scale perception within VSS.	Multi-scale handling is block-internal, not explicit.	Contrast: MACVM-UNet uses a dedicated MSAFA module for explicit bottleneck aggregation.
LightM-UNet [24]	Lightweight Mamba block design.	Fewer parameters, computationally efficient.	Lightweight focus may trade-off feature richness.	Contrast: MACVM-UNet balances performance and efficiency.
VM-UNet (Baseline)	Standard VSS block, unidirectional scan.	First integration of SSM into U-Net for linear-complexity global modeling.	Unidirectional scan may lead to incomplete context; standard skip connections.	Starting point for improvement: MACVM-UNet addresses its shortcomings via three core innovations.
MACVM-UNet	CVSS, CASC, MSAFA	Integrated Solution: CVSS, CASC, MSAFA	As a novel integrated model, requires ablation studies (as in Sec. 4.4).	This work, synthesizing strengths from above to provide a tailored solution for SPWDD.

Although current VSS effectively address the complexity issue of Transformers, they often fail to fully utilize the local spatial background and the complex multi-scale feature fusion. MACVM-UNet fills this deficiency by integrating CVSS, CASC, and MSAFA, aiming to achieve a more comprehensive and effective balance between global context modeling and local detail preservation in SPWDD.

## 3. The proposed model

**Fig 1:**
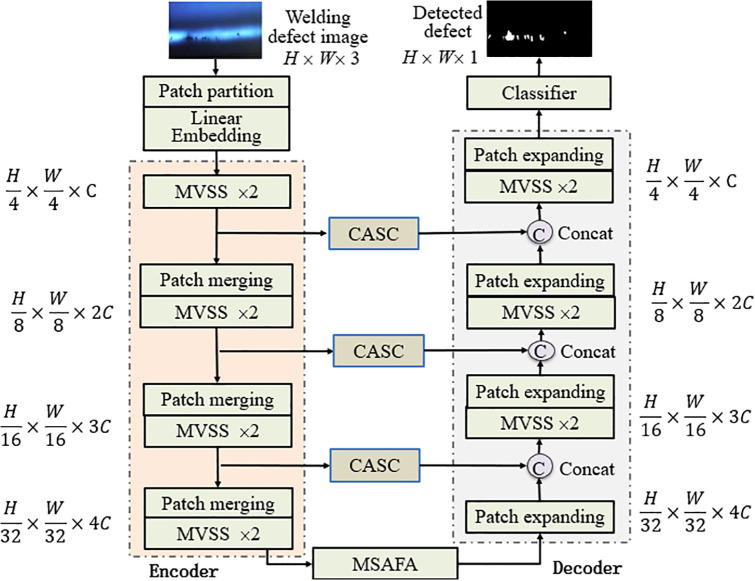
Overview architecture of MACVM-UNet.

The architecture of MACVM-UNet is demonstrated in Fig 1, consisting of patch partition and linear embedding layer, encoder, decoder, classifier, CASC and MSAFA, where the encoder is composed of four stages with 4 CVSS blocks and 3 patch merging blocks, the decoder consists of four stages with 3 CVSS blocks and 4 patch expanding blocks, and classifier is Softmax classifier.

### 3.1 Overall description

First, in patch partition and linear embedding layer, the original input image $Img\in R^{H\times W\times 3}$ is divided into non-overlapping patches of size 4 × 4, and mapped into the C-dimension feature space by linear embedding with *C* default being 96. This process yields the embedded image $Img^{\prime }\in R^{H/4\times W/4\times C}$. In the encoder, the *C*-dimensional tokenized input with a resolution of *H/*4 × *W*/4 × *C* is input into two consecutive CVSS blocks (CVSS×2) for feature representation, where the feature dimension and resolution remain unchanged. The patch merging is repeated three times to reduce the number of tokens (2 downsamplings) and increase the feature dimension to 2 of the original dimensions. Through 4 CVSS layers and 3 patch merging layers with the channel counts for each stage being [C,2C,4C,8C], the output dimensions of 4 stages in the encoder are W/4×H/4×C, W/8×H/8×2C, W/16×H/16×4C and W/32×H/32×8C, respectively.

Compared with patch merging in the encoder, patch expanding in the decoder is replaced four times to upsample the extracted deep features. It reconstructs the feature mapping of adjacent dimensions into a feature mapping of higher resolution (2-upsampling), and accordingly reduces the feature dimension to half of the original dimension. The output dimensions of 4 stages in the decoder are W/32×H/32×8C, W/16×H/16×4C, W/8×H/8×2C and W/4×H/4×C, respectively.

Similar to U-Net, CASC is regarded as Skip-connection, fusing the multi-scale features and upsampling features of the encoder, and concatenating the shallow features and deep features together, reducing the spatial information loss caused by downsampling.

Similar to U-Net, CASC is used as skip-connection to fuse the multi-scale features and upsampling features from the encoder, concatenating the shallow features and deep features together, and reducing the spatial information loss caused by downsampling. Followed by a linear block, the dimension of the concatenated features remains the same as that of the upsampled features. Since MACVM-UNet is too deep to converge, MSAFA is introduced into the bottleneck to learn the deep feature representation while keeping the feature dimension and resolution unchanged.

#### 3.2 Components of MACVM-UNet.

**Fig 2:**
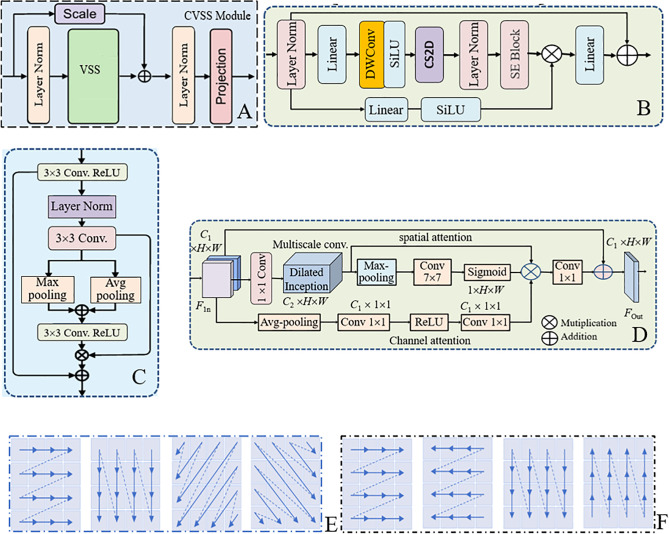
The modular structure of MACVM-UNet. (A) CVSS, (B) VSS, (C) CASC, (D) MSAFA, (E) CS2D, (F) SS2D.

From Fig 1, it is seen that MACVM-UNet is an improved VM-UNet model. Compared with VM-UNet, the improvements of MACVM-UNet include CVSS, CASC, and MSAFA, corresponding to VSS, Skip connection and bottleneck of VM-UNet, respectively. Their structures are shown in Fig 2, and are described as follows.

(1) **CVSS**. CVSS is the basic module in both the encoder and decoder, playing a crucial role in extracting global contextual feature and long-range dependencies. Its structure is shown in Fig 2A, and the structure of its VSS in Fig 2B, where a scale-residual connection is incorporated to generate the fused feature to further improve the feature extraction and representation of SPWDD, the projection block is utilized to transform the fused feature into a deeper feature. The process of CVSS is described as follows,

$$F_{CVSS}=Prj(LN(CVSS(LN(F_{CVSS}^{in}))⊕\lambda \cdot F_{CVSS}^{in}))$$ (1)

where $F_{CVSS}^{in}$ and $F_{CVSS}$ are the input and output, *LN*(.) is the layer normalization operation, *CVSS*(.) is the CVSS operation, *Prj*(.) is the CVSS operation, λ is an adjustment factor.

CVSS operation is described as follows,


$$F_{i}^{\prime }=C_{2}(LN(CS2D(SiLU(DWConv(C_{1}(F_{i}))))))$$ (2)

where $F_{i},F_{i}^{\prime }$ are the input and output feature maps of CVSS in the *i*th stage, respectively, *C*_1_(·) and *C*_2_(·) are two linear projections used to double the channel dimension, *DWConv*(·) is a depth-wise convolution with a kernel size of 3 × 3, *SiLU*(*x*) = *x*·sigmoid(*x*) is an activation function, *CS2D*(·) is cross 2D scanning version.

(2) **CASC**. The original Skip connection in VM-UNet ignores the differences between thefeatures and assigns the same weight to all features, which limits the complementarity of features at different levels from being fully utilized. CASC is regarded as an attention skip-connection with minor modification. Its structure is shown in Fig 2C. It is composed of 3 × 3 Conv, Max- and Avg-pooling, Layer norm, ReLU(the Rectified Linear Unit), two parallel Avg-Max pooling, and two residual connections. Its process is as follows,

$$\begin{array}{c} F_{CASC}^{out}=ReLU(Conv(Avg(F_{LN}),Max(F_{LN})))⊗F_{LN}+F_{CR}\\ F_{LN}=Conv(LN(F_{CR}))\\ F_{CR}=ReLU(Conv(F_{CASC}^{in})) \end{array}$$ (3)

where $F_{CASC}^{in}$ and $F_{CASC}^{out}$ are the input and output of CASC, *Max*(.) and *Avg*(.) are Max- and Avg-pooling operation, *Conv*(·) is 3 × 3 Conv, and *ReLU* (·) is an activation operation.

Different from the standard skip-connections that simply concatenate or add features, CASC can adaptively select the channel representation, reducing the semantic gap between multiscale encoder features and decoder, the Avg-Max pooling operations capture semantic representations. Combining Avg-Max pooling features allows the skip-connection to transmit both low-level texture and high-level semantic features more effectively.

(3) **MSAFA**. To enhance the ability to extract diverse defect features at different spatial scales, MSAFA is introduced to the bottleneck layer of the model. Its structure is shown in Fig 2D, consisting of a spatial attention, a channel attention, two residual connections and a 1 × 1 convolution. Two attentions independently process the input feature map, enabling the module to extract both fine- and coarse-grained contextual information. Spatial attention includes a 1 × 1 convolution, a dilated Inception, parallel Max-pooling and Avg-pooling, residual connection, 7 × 7 convolution, followed by Sigmoid activation and 1 × 1 convolution. Channel attention includes Avg-pooling, two 1 × 1 convolutions, and a ReLU activation. The dilated Inception with dilated rates of 1, 2 and 3 are used to capture multiscale features. Adding the results of the spatial path and the channel path at the element level to obtain the final output feature map. The process of MSAFA is expressed as follows,

$$\begin{array}{c} F_{Out}=Conv_{1\times 1}(F_{Spatial}⊗F_{Channel}⊗F_{Dil})⊕F_{In}\\ F_{Spatial}=\sigma (Conv_{7\times 7}(Max(F_{Dil})))\\ F_{Channel}=Conv_{1\times 1}(ReLU(Conv_{1\times 1}(Avg(F_{In}))))\\ F_{Dil}=Conv_{3\times 3}^{r=1}(F_{In})⊕Conv_{5\times 5}^{r=2}(F_{In})⊕Conv_{7\times 7}^{r=3}(F_{In}) \end{array}$$ (4)

where $F_{In}$ and $F_{Out}$ are the input feature maps and output feature maps of MSAFA, respectively, $F_{Dil}$ is the output of the dilated Inception convolution, σ is the Sigmoid function,$Conv_{p\times p}$ is a convolution with an p×p kernel, $Conv_{3\times 3}^{r=1},Conv_{5\times 5}^{r=2},Conv_{7\times 7}^{r=3}$ are three dilated convolution operations with dilated rates 1,2,3, respectively, ⊕ and ⊗ are concatenation and element-wise multiplication.

In MSAFA, the dilated Inception at multiple rates allows the model to capture local texture (e.g., tiny defect) and global structure (e.g., large defect) simultaneously, which is essential for recognizing welding defects with varying morphology and scale. The final 1 × 1 convolution projects the aggregated features back to the original channel dimension, producing the refined output $F_{Out}$.

(4) **CS2D**. CS2D (2D-Cross-Scan) in CVSS is an improved version of VSS of VMamba. It is a more essential and flexible implementation of SS2D (2D-Selective Scan). Its structure is depicted in Fig 2E, consisting of four paths: horizontal, vertical, diagonal and antidiagonal. For comparison, Fig 2F presents the structure of SS2D, consisting of four paths: horizontal, vertical and two reverses. Compared Fig 2E and Fig 2F, it is known that CS2D can fully capture global information from multiple directions, which is suitable for SPWDD. CS2D captures long-range dependencies in each direction via the selective state-space model, and the direction sequences are concatenated to recover the 2D structure.

### 3.3 Model training

The model is trained using a combined loss function *Loss* that addresses both pixel-wise classification and region overlap for the imbalanced defect pixels, calculated as follows,


$$\begin{array}{c} Loss=\alpha L_{dice}+(1-\alpha )L_{cross}\\ L_{dice}=1-2\sum {\mathrm{\backslash nolimits}}_{i=1}^{H}\sum {\mathrm{\backslash nolimits}}_{j=1}^{W}Gt_{ij}\cdot Pre_{ij}/(\sum {\mathrm{\backslash nolimits}}_{i=1}^{H}\sum {\mathrm{\backslash nolimits}}_{j=1}^{W}Gt_{ij}^{2}+\sum {\mathrm{\backslash nolimits}}_{i=1}^{H}\sum {\mathrm{\backslash nolimits}}_{j=1}^{W}Pre_{ij}^{2})\\ L_{cross}=-\frac{\text{1}}{N}\sum {\mathrm{\backslash nolimits}}_{i=1}^{H}\sum {\mathrm{\backslash nolimits}}_{j=1}^{W}\sum {\mathrm{\backslash nolimits}}_{c=1}^{2}y_{ij}^{c}\log{\tilde{y}}_{ij}^{c} \end{array}$$ (5)

where $L_{dice}$ and $L_{cross}$ are Dice loss and cross-entropy loss, *H* and *W* are the height and width of the feature map, respectively, $Gt_{i,j}$ and $Pre_{i,j}$ are the corresponding binary labels (0 for background, 1 for defect) for the Loss at the position (*i, j*), $y_{ij}^{c}$ and ${\tilde{y}}_{ij}^{c}$ are the labels of the Ground Truth and the predicted pixel of defect image at the *i*-th and *j-*th position, respectively, α is an adjustment coefficient, and the default value is 0.45.

## 4. Experiments and analysis

To demonstrate the MACVM-UNet-based SPWDD method, a large number of experiments are conducted on the WDXI dataset, and compared with a baseline network U-Net and five the-state-of-the-art SPWDD methods, such as attention U-Net (AU-Net) [17], CGTD-Net [18], TransUNet [19], VM-UNet [10], VM-UNet++ [11], MSVM-UNet [23], LightM-UNet [24]. These methods are briefly described as follows.

U-Net is an encoder-decoder architecture that uses convolutional layers to extract more robust high-level semantic features.

AU-Net introduces attention mechanism into skip connection to improve the accuracy of defect detection.

CGTD-Net is a dual-branch network based on the channel-wise global Transformer, using the Spatial Channel Global Concern (SCGA) module to enhance the feature extraction of spatial channels.

TransUNet is a hybrid UNet by incorporating Transformer encoder to improve the ability to extract defect features in complex environments.

VM-UNet is a modified U-Net by integrating Vision Mamba into U-Net.

VM-UNet++ is an advanced nested VM-UNet for precise medical image segmentation.

MSVM-UNet is a Multi-Scale VM-UNet model by introducing multi-scale convolutions in VSS to capture and aggregate multi-scale feature representations from the hierarchical features of the VMamba encoder and better handle 2D visual data.

LightM-UNet integrates Mamba and UNet in a lightweight framework, utilizing the residual visual Mamba layer to extract deep semantic features and model long-range spatial dependencies, while has linear computational complexity.

All the above models are implemented by DL framework PyTorch and Win10 operating system: Intel Core i7 3.2GHZ processor with NVIDIA GeForce GTX1060 GPU, PyTorch 1.12.1, and CUDA 11.6. Four metrics mean-Accuracy (*mAcc*), mean-Precision (*mPre*), mean-Recall (*mRec*) and mean-F1(*m*F1) are commonly adopted to measure the effect of DL models, calculated as follows,


$$\begin{array}{c} mAcc=\frac{\sum T_{P}+\sum T_{N}}{\sum T_{P}+\sum F_{P}+\sum T_{N}+\sum F_{N}}\mathrm{\backslash vspace}1.5mm\\ mPre=\frac{\sum T_{P}}{\sum T_{P}+\sum F_{P}}\mathrm{\backslash vspace}1.5mm\\ mRec=\frac{\sum T_{P}}{\sum T_{P}+\sum F_{N}}\mathrm{\backslash vspace}1.5mm\\ mIOU=\frac{\sum T_{P}}{\sum T_{P}+\sum F_{P}+\sum F_{N}}\mathrm{\backslash vspace}1.5mm\\ mF1=\frac{2PR}{P+R} \end{array}$$ (6)

where the meanings of *T*_*P*_, *F*_*P*_, *F*_*N*_ and *T*_*N*_ are indicted as in Table 4, $\sum T_{P}$, $\sum F_{P}$, $\sum T_{N}$ and $\sum F_{N}$ are the pixel numbers of true positive, false positive, true negative and false negative, respectively, *F*1 is used to measure precision and recall.

**Table 4:** The meaning of four variables.

True value Predicted value	True positive	True negative
Predicted positive	*T*_*P*_	*F*_*P*_
Predicted negative	*F*_*N*_	*T*_*N*_

The essential efficiency metrics alongside performance scores are used as metrics to measure the performance of each model.

- Number of Parameters (Params): Measured in millions (M), indicating model size.
- Computational Complexity (GFLOPs): Measured in Giga-FLOPs for a standard input size (e.g., 256 × 256), indicating theoretical forward-pass computational cost.
- Training Time (h): Retained from the original, indicating training efficiency.

To ensure reproducibility, all experiments are conducted with PyTorch built-in and NumPy random seeds fixed to 42. The reported average training time for each model in Table 5 is computed as the mean total wall-clock time (in hours) taken to complete the full 300-epoch training process across all five folds of the cross-validation. This measurement is performed on the specified hardware (RTX2070 GPU) with no other competing intensive processes running. All compared models are trained under this identical protocol.

**Table 5:** The distribution of defect types of the dataset.

Defect type	Number of images	Description
Porosity (PO)	500	Clustered or scattered small, circular dark spots.
Slag Inclusion (SI)	98	Irregular, elongated dark streaks or patches.
Lack of Fusion (LF)	152	Fine, linear discontinuities along weld edges.
Incomplete Penetration (IP)	180	Continuous linear dark lines at the weld root.
Cracks (CR)	70	Thin, sharp-contoured linear indications, often branched.

### 4.1 Datasets

#### 4.1.1 GDXray.

The WDXI dataset GDXray (https://domingomery.ing.puc.cl/material/gdxray) contains 1,000 welding defect images, covering five types of defects: incomplete welding, porosity, slag inclusion, incomplete fusion and cracks. Half of the images are from GDXray [26,27], and the other half are from the pipe weld inspection results of Shandong Shengli Steel Pipe Co., Ltd. in the past three years. The distribution of defect types is unbalanced, with 500 porosity images and 98 slag images, and the marking of each type is biased towards porosity. The detail distribution of defect types of the dataset is shown in Table 5.

**Fig 3:**
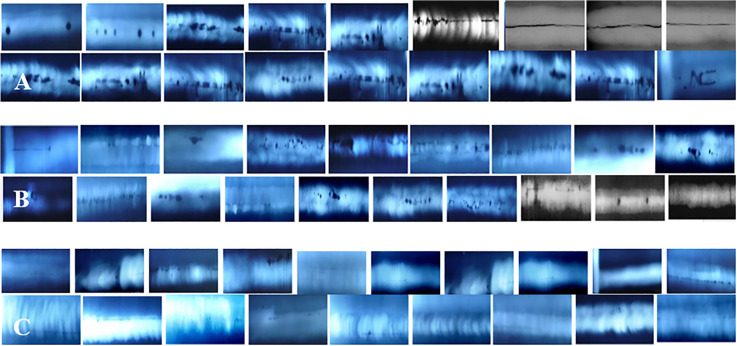
Original WDXIs, and cropped images in GDXray. **(A)** Obvious defects, **(B)** Not obvious and tiny defects, **(C)** Fuzzy defects.

Some examples are shown in Fig 3A, 3B and 3C. From Fig 3, it is seen that there are various defects, such as circular, linear, black lines or strips of varying width, discontinuous or continuous black lines with neat contours, and sharp-contoured black lines, and some defect images cannot be distinguished from the background by the naked eye, as seen in Fig 3C. The occurrence frequency of different types of weld defects is unbalanced. The WDXIs are complex and diverse, with different sizes and shapes, variable cracks, blurred edges and patterns, relatively small bubble defects, and low contrast of unfused defects, which cannot be fully detected by traditional ML and DL.

#### 4.1.2 RIWD.

**Fig 4:**
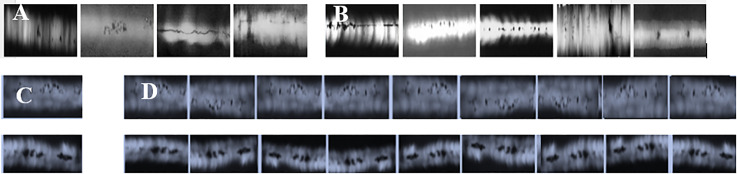
Original WDXIs and augmented images in RIWD. **(A)** R0001, **(B)** R0002, **(C)** Cropped image, **(D)** Augmented images.

The Radiographic Images of Welding Defects (RIWD) is a cross-dataset. It comprises two purpose-built subsets: R0001 (57 images of illustrative plates with obvious, numerous, and diverse defects) to effectively train models in learning discriminative defect features, and R0002 (59 images of scanned real weld radiographic films containing rare, small, and subtle defects) to rigorously test the model’s generalization and detection capability in challenging, realistic scenarios. This two-part structure allows for comprehensive evaluation, from learning performance to practical robustness. Fig 4 shows some samples in RIWD.

#### 4.1.3 Data augmentation strategy.

To improve generalization and overcome overfitting, an extensive data augmentation strategy is employed. Each original image in the training set is augmented by a factor of nine through a series of random transformations, yielding a final augmented dataset of 10,000 images (1,000 originals and 9,000 augmented samples). This process enhances the diversity of welding defect appearances, as follows:

Random Rotation: Within a range of [−15°, + 15°].

Random Brightness & Contrast Adjustment: Brightness and contrast scaling factors both sampled from [0.8, 1.2].

Horizontal & Vertical Flipping: Each applied with a probability of 0.5.

Random Gaussian Noise Addition: Applied with a probability of 0.3, where the standard deviation is sampled from [0.01, 0.05].

Images in the test sets are only subjected to standardization (mean subtraction and standard deviation division), with no augmentation applied.

#### 4.1.4 Data split and reproducibility.

To ensure strict and repeatable evaluation, a five-level cross-validation (FFCV) strategy is adopted. First, a fixed random seed is used to shuffle the entire augmented dataset of 10,000 images. Then it is divided into five non-overlapping subsets of equal size (2000 images in each subset). In each fold, four subsets are used for training and the remaining one for testing. This process is repeated five times so that each subset is used as a test set once. Save these partitioned indexes to ensure that all experiments and comparisons are conducted on the same data partition.

#### 4.2 Experiment setting.

All experiments are implemented with PyTorch 1.13.0 and conducted on a workstation with an NVIDIA RTX 4090 GPU. The model is trained from scratch for 300 epochs using a combined Dice and Cross-Entropy loss (λ = 0.45), the AdamW optimizer with an initial learning rate of 1e-3, a cosine annealing scheduler, a weight decay of 0.01, and a batch size of 8. On-the-fly data augmentation, including flipping, rotation (±15°), and random brightness/contrast adjustments. Key configurations are summarized in Table 6.

**Table 6:** The grid-search results of hyperparameters in FFCV-experiments.

Parameter	Value/ Setting	Rationale/ Comment
Total Epochs	300	Determined by convergence on validation set.
Batch Size	8	Maximized based on GPU (RTX 4090) memory.
Optimizer	AdamW	With betas=(0.9, 0.999).
Initial Learning Rate	1e-3	Standard starting point for AdamW.
LR Scheduler	Cosine Annealing	Decays LR to a minimum of 1e-5.
Weight Decay	0.01	For regularization.
Composition	$Loss=\alpha L_{dice}+(1-\alpha )L_{cross}$	Combines region and pixel-wise terms.
Dice Weight (λ)	0.45	Empirically tuned for the WDXI dataset.
Flipping	Horizontal & Vertical (p = 0.5)	Increases spatial invariance.
Rotation	±15 degrees	Accounts for variable orientations.
Brightness/Contrast	Scale: [0.8, 1.2]	Simulates varying exposure.
Gaussian Noise	σ∈[0.01, 0.03] (p = 0.2)	Improves robustness to sensor noise.
GPU	NVIDIA GeForce RTX 4090 (24GB)	For processing high-resolution images.
Framework	PyTorch 1.13.0, CUDA 11.7	

**Fig 5:**
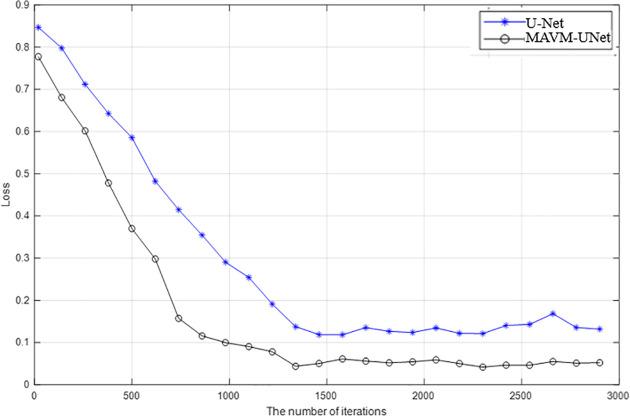
Loss of *mAcc* of MACVM-UNet and U-Net versus the number of iterations.

Fig 5 is the Loss of *mAcc* of MACVM-UNet and U-Net versus the number of iterations. From Fig 5, it is seen that the convergence performance of MACVM-UNet is obviously better than that of U-Net, while the loss curve of U-Net fluctuated greatly, and their losses decrease rapidly before the 1500th iteration and tend to be stable after the 2500th iteration. After 3000 iterations, two models basically converge. To be fair, in the following experiments, all models are trained through 3000 iterations.

### 4.3 Defect detection results

**Fig 6:**
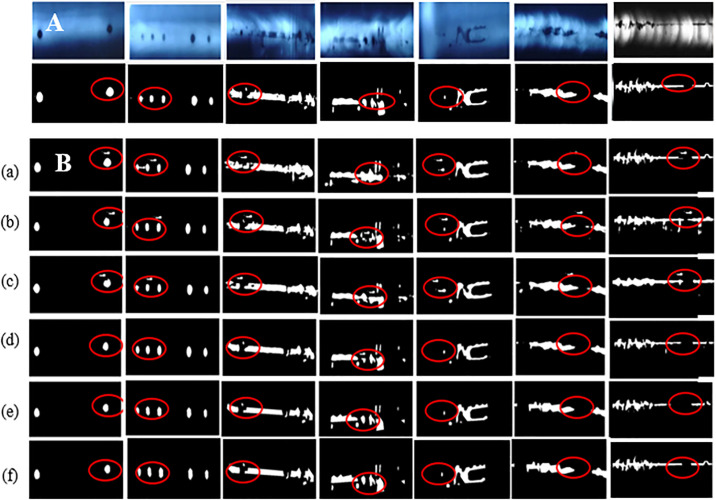
(A) Original simple obvious defect images and corresponding labeled images, (B) Detected defect images.Comparison detection results of simple defect images by 8 models, where (a) AU-Net, (b) CGTD-Net, (c) TransUNet, (d) VM-UNet, (e) VM-UNet++, (f) MSVM-UNet, (g) LightM-UNet, and (h) MACVM-UNet.

AU-Net [17], CGTD-Net [18], TransUNet [19], VM-UNet [10], VM-UNet++ [11], MSVM-UNet [23], LightM-UNet [24] and MACVM-UNet are trained and tested by FFCV using the same dataset and experimental environment. Fig 6 illustrates the detected defects of 7 simple defect images. From Fig 6A, it is observed that the defects are obvious with different proportions and clear shapes of defects. From Fig 6B, it is seen that the locations and shapes of defects can be accurately detected by 8 models, and there is little difference between all models, and MACVM-UNet is close to the corresponding labeled images, particularly in the discontinuous black line defects, while AU-Net is worse having some small misclassified regions.

**Fig 7:**
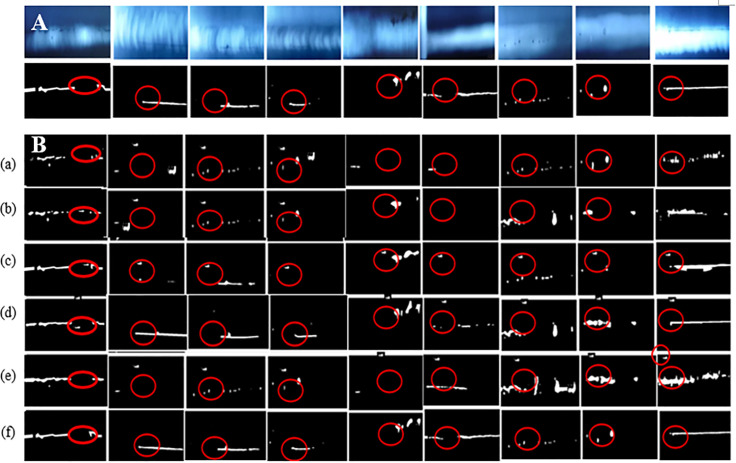
(A) 7 complex original WDXIs and corresponding annotated images, (B) Detected defect images.Comparison detection results of complex defect images by 8 models, where (a) AU-Net, (b) CGTD-Net, (c) TransUNet, (d) VM-UNet, (e) VM-UNet++, (f) MSVM-UNet, (g) LightM-UNet, and (h) MACVM-UNet.

To compare the feasibleness, Fig 7B shows the detected defects of 7 complex WDXIs by 8 models. From Fig 7A, it is seen that the defects are blurred with no clear boundary. It is very difficult for the naked eye to accurately and completely distinguish defects from the welding images.

From Fig 6B, it is found that, compared with other models, MACVM-UNet can detect very small fuzzy defects, and the defects detected by MACVM-UNet are closer to the corresponding labeled defects. AU-Net and CGTD-Net will generate large errors and over-split or over-split the defect images, while MACVM-UNet and MSVM-UNet can detect the defect images better.

To quantitatively evaluate the detection performance of MACVM-UNet, a large number of extensive SPWDD experiments are conducted through FFCV. Table 7 shows the average detection results and the training time of 8 methods.

**Table 7:** Comprehension of model performance and efficiency on the WDXI dataset.

Model	Params (M) ↓	GFLOPs ↓	mIoU (%) ↑	mPre (%) ↑	mRec (%) ↑	mF1 (%) ↑	Training Time (h) ↓
AU-Net [17]	39.0	245.3	78.4	81.76	79.65	80.69	1.15
CGTD-Net [18]	–	–	–	83.73	82.69	83.20	2.94
TransUNet [19]	105.6	512.8	79.0	82.52	81.12	81.81	3.64
VM-UNet [10]	29.7	95.8	80.1	83.85	82.70	83.27	1.58
VM-UNet++ [11]	35.1	118.6	81.0	85.05	83.90	84.47	1.92
MSVM-UNet [23]	33.8	105.2	80.2	84.46	83.75	84.10	1.85
LightM-UNet [24]	**28.5**	**41.7**	76.1	83.70	82.54	83.11	**0.84**
MACVM-UNet	34.2	112.5	**81.7**	**86.14**	**83.12**	**84.60**	1.61

From Table 7, it is seen that MACVM-UNet achieves the highest scores across three critical metrics (mIoU: 81.7%, mPre: 86.14%, mF1: 84.60%), establishing itself as the top-performing model in this comprehensive comparison. A clear performance trend is observed among Mamba-based models:

VM-UNet serves as the baseline with solid performance (mF1: 83.27%). MSVM-UNet improves upon this (mF1: 84.10%) through multi-scale feature handling. VM-UNet++ shows further enhancement (mF1: 84.47%). MACVM-UNet outperforms all with the highest mF1 of 84.60%, demonstrating the effectiveness of our integrated CVSS, CASC, and MSAFA modules. CGTD-Net, LightM-UNet, TransUNet, and UI-Net, UI-Net has blurred defect contours and minor defect losses. The training time order of the six models is as follows: LightM-UNet is the fastest, followed by AU-Net, MSVM-UNet, MACVM-UNet, CGTD-Net, and TransUNet is the slowest. LightM-UNet is the most lightweight (28.5M params, 0.84h training), it sacrifices performance (lowest mIoU: 76.1%). MACVM-UNet achieves superior performance with only 34.2M parameters and 1.61h training time, offering an excellent balance between accuracy and efficiency.

MACVM-UNet is an improved MSVM-UNet. Table 8 shows the comparative results of MACVM-UNet and MSVM-UNet on the WDXI test set. Values are mean ± standard deviation across 5 folds. Best scores are in **bold**.

**Table 8:** The results of MACVM-UNet and MSVM-UNet.

Defect category	Model	mPre (%)	mRec (%)	mF1 (%)	mIoU (%)
Porosity (PO)	MSVM-UNet	92.1 ± 0.8	90.3 ± 1.1	91.2 ± 0.7	83.9 ± 1.0
	MACVM-UNet	**93.5 ± 0.6**	**92.0 ± 0.9**	**92.7 ± 0.5**	**86.4 ± 0.8**
Slag Inclusion (SI)	MSVM-UNet	81.5 ± 2.1	78.2 ± 2.5	79.8 ± 1.9	66.5 ± 2.3
	MACVM-UNet	**84.7 ± 1.7**	**82.0 ± 2.0**	**83.3 ± 1.5**	**71.4 ± 2.0**
Lack of Fusion (LF)	MSVM-UNet	83.2 ± 1.5	80.1 ± 2.0	81.6 ± 1.4	69.0 ± 1.8
	MACVM-UNet	**85.8 ± 1.2**	**84.5 ± 1.6**	**85.1 ± 1.1**	**74.0 ± 1.5**
Incomplete Penetration (IP)	MSVM-UNet	88.7 ± 1.0	85.4 ± 1.4	87.0 ± 0.9	77.0 ± 1.2
	MACVM-UNet	**90.2 ± 0.8**	**88.9 ± 1.1**	**89.5 ± 0.7**	**81.0 ± 1.0**
Cracks (CR)	MSVM-UNet	76.8 ± 3.0	72.5 ± 3.5	74.6 ± 2.8	59.8 ± 3.3
	MACVM-UNet	**80.1 ± 2.5**	**77.8 ± 3.0**	**78.9 ± 2.4**	**65.3 ± 2.9**

As shown in Table 8, MACVM-UNet achieved the best average performance in all evaluation indicators of all five types of defects, especially showing significant improvements in key defects such as cracks and slag inclusions that are difficult to detect (mIoU increased by 5.5% and 4.9% respectively). MACVM-UNet demonstrated lower standard deviations in all results (for instance, the standard deviation of the mF1 score for cracks decreased from ±2.8% to ±2.4%). This indicates that its performance is more stable and reliable on different data subsets, further verifying that the model has stronger robustness and generalization ability. Therefore, MACVM-UNet not only achieves higher average detection accuracy, but also has more consistent prediction results, providing solid evidence for the effectiveness of the proposed CVSS, CASC and MSAFA modules.

To objectively validate that the performance improvements of MACVM-UNet are statistically significant and not due to random variation, a paired t-test (two-tailed, *α* = 0.05) is conducted on the mF1 scores obtained from the five cross-validation folds. The results, presented in Table 9, compare MACVM-UNet against each baseline model. The null hypothesis (H₀) states that there is no difference in mean performance between the two compared models.

**Table 9:** Statistical significance analysis (Paired t-test on mF1 scores).

Comparison	mF1 Difference	p-value	p < 0.05
MACVM-UNet vs. MSVM-UNet	+0.50%	0.018	Yes
MACVM-UNet vs. LightM-UNet	+1.49%	0.003	Yes
MACVM-UNet vs. CGTD-Net	+1.40%	0.005	Yes
MACVM-UNet vs. TransUNet	+2.79%	<0.001	Yes
MACVM-UNet vs. AU-Net	+3.91%	<0.001	Yes

The results in Table 9 confirm that the superior performance of MACVM-UNet is statistically significant for all baseline models. Notably, the p-value of 0.018 for the comparison with MSVM-UNet, its closest competitor, provides strong evidence to reject H₀ and supports the claim that our architectural modifications lead to a genuine performance enhancement.

### 4.4 Ablation experiments

MACVM-UNet is an improved U-Net or VMU-Net, different from U-Net and VMU-Net in encoding part, decoding part, bottleneck part and skip-connection. To evaluate the effect of some simplified versions of MACVM-UNet by replacing its components with the components of U-Net and VMU-Net, a number of FFCV experiments are carried out. The WDXI results of the simplified versions of MACVM-UNet are shown in Table 10, Row (7) replaces the MSAFA module with a Convolutional Block Attention Module (CBAM) for comparison.

**Table 10:** The WDXI results of the simplified versions of MACVM-UNet.

Results Model Variant	CVSS	CASC	MSAFA	mAcc (%) ↑	mF1 (%) ↑	mIoU (%) ↑
(1) VM-UNet (Baseline)	×	×	×	81.45	80.50	68.32
(2) Baseline + CVSS	√	×	×	83.15	82.25	70.18
(3) Baseline + CASC	×	√	×	82.53	81.60	69.55
(4) Baseline + MSAFA	×	×	√	82.27	81.35	69.28
(5) Baseline + CVSS + CASC	√	√	×	84.92	83.95	73.41
(6) Baseline + CVSS + MSAFA	√	×	√	85.41	84.58	74.20
(7) MSAFA → CBAM	√	√	(CBAM)	85.27	84.05	73.98
(8) MACVM-UNet(Full Model)	√	√	√	86.03	84.60	75.10

Table 10 shows the contribution of each module to the performance of the final model. From the VM-UNet baseline (81.45% *mAcc*), each of CVSS, CASC and MSAFA provides measurable *mAcc*, and CVSS offers the most significant single-module improvement (+1.70% *mAcc*), while combining these components yields performance superior to the sum of their individual contributions, revealing a powerful synergistic effect, where CVSS+CASC achieves a *mAcc* of 84.92%, exceeding the expected additive results. The complete MACVM-UNet model integrates these three innovations and achieves a peak *mAcc* performance of 86.03%, indicating that the coordinated operation of CVSS, CASC and MSAFA is optimal. When MSAFA is replaced with standard CBAM modules, the observed performance degradation further verifies the effectiveness of MACVM-UNet.

**Fig 8:**
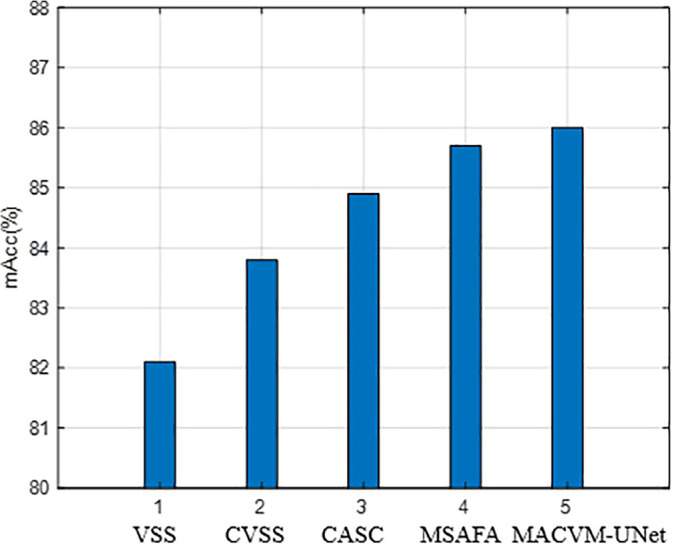
Contribution of individual modules to model performance (mAcc).

Fig 8 presents a bar chart quantifying the impact of each module on mAcc by systematically removing or replacing components in the full MACVM-UNet model. The baseline “VSS” refers to a model using the standard VSS block without our modifications.

The results demonstrate that each module provides a cumulative performance gain. The **CVSS** module offers the most substantial single improvement (+1.7%), highlighting the critical importance of comprehensive multi-directional context capture for SPWDD. The three core components of MACVM-UNet: CVSS, CASC, and MSAFA—exhibit a synergistic, rather than merely additive, interaction that is central to the model’s superior performance. The comprehensive, multi-directional global context captured by **CVSS** establishes a coherent feature foundation. This enriched context makes the feature channels more informative, thereby enhancing the effectiveness of CASC adaptive channel re-weighting for precise detail recovery. Simultaneously, this well-structured global feature set serves as a superior input for MSAFA at the bottleneck, enabling more meaningful multi-scale aggregation. In turn, the refined multi-scale features from MSAFA and the calibrated high-resolution details from CASC create a virtuous cycle, where rich contextual information flows bidirectionally during decoding. This integrated mechanism ensures that the model excels at segmenting defects that require both long-range dependency understanding and precise multi-scale localization.

To verify the effectiveness of CS2D, five variants of the CVSS block is employed for SPWDD by selectively enabling different combinations of its four scanning paths (Horizontal-H, Vertical-V, Diagonal-D, Anti-diagonal-AD). Each variant is integrated into the MACVM-UNet architecture and retrained on the WDXI dataset under identical conditions. The results of mAcc and mF1 are presented in the Table 11.

**Table 11:** Performance of CVSS variants with different scanning path combinations.

Scanning path configuration	mAcc (%)	mF1 (%)
H + V (Standard SS2D)	84.92	83.95
H + V + D	85.41	84.58
H + V + AD	85.38	84.55
H + V + D + AD (CS2D)	86.03	84.60
D + AD	83.15	82.01

The results in Table 11 verifies that CS2D is effective as the optimal configuration. The experimental performance (86.03% mAcc) not only significantly outperforms the standard dual-channel SS2D scheme (84.92% mAcc), but more importantly, it demonstrates its inherent advantages.

### 4.5 Experiments on cross-dataset

To verify the generalization, a lot of experiments are conducted on a cross-dataset RIWD to verify our MACVM-UNet model. This is critical to prove its robustness against real-world domain shifts. Table 12 shows the comparative results on **RIWD R0002 test set**

**Table 12:** The comparison of different models on the RIWD R0002 test set.

Model	mPre (%) ↑	mRec (%) ↑	mF1 (%) ↑	mIoU (%) ↑
**MACVM-UNet**	**79.1**	**78.7**	**78.9**	**65.4**
MSVM-UNet	75.6	74.7	75.1	61.8
Attention U-Net	73.5	70.6	72.0	58.2
U-Net	71.2	69.9	70.5	55.7

The cross-dataset evaluation on the challenging RIWD R0002 test set provides strong evidence for the superior generalization ability of our proposed MACVM-UNet. From Table 12, it is found that **MACVM-UNet** achieves the best performance across all metrics, with a notable mIoU of 65.4% and a balanced mF1-score of 78.9%. Its leading recall rate of 78.7% indicates a stronger capability to detect subtle and rare real-world defects, which is critical for reliable weld inspection.

## 5. Conclusions

Multi-attention Cross-Scan VM-UNet (MACVM-UNet) is constructed to solve the problem of detecting welding defects in steel pipes. It consists of three collaborative components: (1) Cross-Scan VSS (CVSS) integrates multiple scan paths to capture comprehensive remote dependencies that are crucial for irregular defects, (2) Channel Attention Skip Connection (CASC) adaptively fuses multi-scale features to refine defect boundaries, and (3) Multi-scale Attention Feature Aggregation (MSAFA) is used to model robust representations of defects of different sizes. The experiment results on the WDXI datasets verify that the proposed model outperforms the state-of-the art methods, and achieves *mAcc* 86.03% and *mPre* of 86.14%, overcoming the limitations of CNNs in global context modeling and the high computational cost of Transformers. The architecture presents a generalizable paradigm with potential applications in broader industrial inspection domains. Future work will focus on model lightweighting for edge deployment and exploring few-shot learning techniques to reduce reliance on annotated data.
